# Understanding acceptability in the context of text messages to encourage medication adherence in people with type 2 diabetes

**DOI:** 10.1186/s12913-021-06663-2

**Published:** 2021-06-28

**Authors:** Y. Kiera Bartlett, Cassandra Kenning, Jack Crosland, Nikki Newhouse, Lisa M. Miles, Veronika Williams, Jenny McSharry, Louise Locock, Andrew J. Farmer, David P. French

**Affiliations:** 1 Manchester Centre for Health Psychology, School of Health Sciences, University of Manchester, Coupland 1 Building, Oxford Road, Manchester, M13 9PL UK; 2 Division of Population Health, Health Services Research & Primary Care, School of Health Sciences, University of Manchester, Manchester, UK; 3 Nuffield Department of Primary Care Health Sciences, University of Oxford, Oxford, UK; 4 School of Nursing, Nipissing University, North Bay, Canada; 5 Health Behaviour Change Research Group, School of Psychology, NUI Galway, Galway, Republic of Ireland; 6 Health Services Research Unit, University of Aberdeen, Aberdeen, UK

**Keywords:** Acceptability, mHealth, Type 2 diabetes, Text messaging

## Abstract

**Background:**

Acceptability is recognised as a key concept in the development of health interventions, but there has been a lack of consensus about how acceptability should be conceptualised. The theoretical framework of acceptability (TFA) provides a potential tool for understanding acceptability. It has been proposed that acceptability measured before use of an intervention (anticipated acceptability) may differ from measures taken during and after use (experienced acceptability), but thus far this distinction has not been tested for a specific intervention. This paper 1) directly compares ratings of anticipated and experienced acceptability of a text message-based intervention, 2) explores the applicability of the TFA in a technology-based intervention, and 3) uses these findings to inform suggestions for measuring acceptability over the lifespan of technology-based health interventions.

**Methods:**

Data were obtained from a quantitative online survey assessing anticipated acceptability of the proposed text messages (*n* = 59) and a 12-week proof-of-concept mixed methods study assessing experienced acceptability while receiving the text messages (*n* = 48). Both quantitative ratings by return text message, and qualitative data from participant interviews were collected during the proof-of-concept study.

**Results:**

The quantitative analysis showed anticipated and experienced acceptability were significantly positively correlated (r_s_ > .4). The qualitative analysis identified four of the seven constructs of the TFA as themes (burden, intervention coherence, affective attitude and perceived effectiveness). An additional two themes were identified as having an important impact on the TFA constructs (perceptions of appropriateness and participants’ role). Three suggestions are given related to the importance of appropriateness, what may affect ratings of acceptability and what to consider when measuring acceptability.

**Conclusions:**

The high correlation between anticipated and experienced acceptability was a surprising finding and could indicate that, in some cases, acceptability of an intervention can be gauged adequately from an anticipated acceptability study, prior to an expensive pilot or feasibility study. Directly exploring perceptions of appropriateness and understanding whether the acceptability described by participants is related to the intervention or the research - and is for themselves or others - is important in interpreting the results and using them to further develop interventions and predict future use.

## Background

The acceptability of an intervention has been identified as one of five key short-term effects of successful development of complex interventions to improve health and healthcare [1]. Despite the recognised importance, acceptability as a concept applied to health-related interventions in general and health-related technology-based interventions in particular, has been ill-defined [2, 3]. There is a well-established literature on the acceptability of new technology, or new uses for existing technology (e.g. the Technology Acceptance Model (TAM [4])). However, a notable omission in models such as the TAM is a lack of recognition that acceptability and acceptance change over the lifespan of an intervention from development, to implementation, to long-term use [3].

**Fig. 1:**
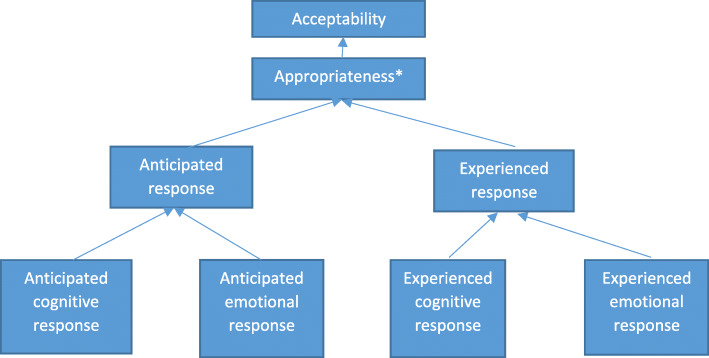
Diagram representing the definition of acceptability [2] * Appropriateness in this case refers to how appropriate either those who receive or deliver a health care intervention consider it to be

More recently attempts have been made to create a definition and framework of acceptability for use across healthcare interventions. Following a review of previous research and a consensus study, the proposed definition of acceptability was: “a multi-faceted construct that reflects the extent to which people delivering or receiving a healthcare intervention consider it to be appropriate, based on anticipated or experienced cognitive and emotional responses to the intervention” [2]. Key aspects of this definition are that i) acceptability relies on an intervention being perceived as appropriate, ii) that appropriateness is based on both cognitive and emotional responses and iii) that acceptability can be assessed prior to use, during use or after using the intervention. Implying that, although the same facets are deemed important, there may be a difference between *anticipated* acceptability measured prior to use and *experienced* acceptability measured during or after use. This definition of acceptability is represented diagrammatically by the current authors in Fig. 1. Although the definition incorporates a temporal element, thus far there has been little research to explore the relationship between anticipated and experienced acceptability measured across the lifespan of a single intervention and what this might mean for those developing interventions.

In addition to the definition, Sekhon et al. [2] propose the Theoretical Framework of Acceptability (TFA) which includes seven constructs thought to influence acceptability; affective attitude, burden, ethicality, intervention coherence, opportunity costs, perceived effectiveness and self-efficacy. Of the 43 systematic reviews used to inform this definition and framework of acceptability however, only four reviews were specifically focussed on technology-based interventions. Of these, two targeted anxiety and depression, one post-traumatic stress disorder and one remote monitoring in primary care. There are therefore unresolved questions about how applicable this framework of acceptability may be to technology-based health care interventions.

To address this, this paper describes studies conducted using a text message-based intervention to encourage medication adherence for people with type 2 diabetes. Worldwide, diabetes is one of the most common long-term conditions affecting 422 million people [5]. Poor adherence to medication regimens, whether intentional or unintentional, are common [6] and lead to poor diabetes control and increased use of health resources [7]. Text messages targeting a range of self-management behaviours have been shown to be effective in reducing blood glucose levels in people with type 2 diabetes [8]. Text messages have the advantage over more complex technologies that they are familiar to most people [9]. However, familiarity with text messages as a mode of delivery does not necessarily imply that a text message-based intervention would be acceptable to the target population as a way of prompting behaviour change.

Prior to the studies reported here, a library of text messages was developed based on relevant behaviour change techniques (BCTs). BCTs are irreducible components of an intervention and have been described as the ‘active ingredients’ of an intervention [10]. A list of 93 BCTs including labels, definitions and examples has been developed including BCTs such as ‘action planning’ and ‘prompts and cues’ [11]. The BCTs used in the messages were identified through a rapid systematic literature review [12] and have all been associated with improved medication adherence in previous studies. Messages were assessed and those deemed unacceptable to the target audience, or that did not represent their intended BCT were removed prior to the proof-of-concept study. This resulted in a library of 290 messages based on 24 BCTs (see Table 1 for example messages). For further details on the development process see [12, 13]. It was important at the outset to ensure no messages deemed unacceptable were included in the library, and applying a yes/no threshold of acceptability ensured this was the case. However, this assessment did not give us an understanding of the multi-faceted nature of acceptability in this context that would allow us to further develop the intervention (if necessary) and to make predictions about future engagement and use.

**Table 1:** Example messages with associated Behaviour Change Technique (BCT) and code from the v1 taxonomy [11]

BCT	Example messages
BCT 1.4: Action Planning	Plan when, where and how you are going to take your medication.
BCT 15.1: Verbal persuasion about capability	If you are struggling with your diabetes tablets then don’t worry, you will be able to master it in time. You will get on top of it.
BCT 7.1: Prompts and cues	It can be difficult to remember to take your tablets. Why not set an alarm to remind you to take them?

The current paper aims to 1) directly compare ratings of anticipated and experienced acceptability of a text message-based intervention to promote medication adherence for people with type 2 diabetes, 2) explore the applicability of TFA in a technology-based health intervention, and 3) use these findings to inform suggestions for measuring acceptability over the lifespan of technology-based health interventions.

## Method

The MRC guidance on developing complex interventions recommends the use of both qualitative and quantitative methods during the development cycles to assess facets such as acceptability [14]. In addition, it has been advised that when designing technology-based health interventions, formative assessments during development are key [15]. Two studies with distinct participants are reported here, an online survey to assess anticipated acceptability of the messages and a proof-of-concept study to assess experienced acceptability. The survey generated quantitative data, the proof-of-concept study generated both quantitative and qualitative data. The analysis of quantitative data from both the online survey and the proof-of-concept study were analysed prior to analysing the qualitative data from the proof-of-concept study, then both sets of findings were used to inform suggestions for the future measurement of acceptability.

### Anticipated acceptability study (quantitative data from an online survey)

#### Design

An online survey study assessed acceptability of 72 text messages associated with 24 BCTs (3 messages per BCT). The acceptability measured was defined as anticipated acceptability as participants did not receive the text messages on their mobile phone but assessed messages as part of a survey.

#### Recruitment

Participants were adults (aged ≥18 years), who self-reported a diagnosis of type 2 diabetes, were taking tablet medication to manage their diabetes (with or without concurrent insulin) and had access to a mobile phone on which they could send and receive messages. Participants were recruited through Research for the Future – Diabetes database (RftF), an NHS-backed database of people who have diabetes, and through face-to-face support groups run by Diabetes UK and independently. Eight hundred and sixty-one people registered with RftF were contacted with information about the study and face-to-face support group facilitators were asked to disseminate the information to their members.

#### Survey development

The survey was designed to assess 72 messages split into two versions of the survey, with 36 messages each. Both surveys were presented with messages in one order, and the reversed order. Participants were randomly assigned to one of the four versions. For each message, participants were asked to rate three facets of anticipated acceptability-cognitive response, emotional response and appropriateness on 5-point Likert scales based on Sekhon et al.’s (2017) definition of acceptability [2]. As we were measuring acceptability per message, we focussed on these key facets included in the definition of acceptability. We asked for a review of 36 messages, and so were keen to reduce participant burden as much as possible. Although the definition allows for appropriateness as considered by those either delivering or receiving a healthcare intervention, for this analysis we have only measured the latter - user perceived appropriateness - as we are interested in acceptability of the messages to the user. Anticipated cognitive response was measured by asking how easy the message was to understand (from ‘very difficult to understand’ to ‘very easy to understand’). Anticipated emotional response was measured by asking how much participants liked the message (from ‘do not like at all’ to ‘like a lot’); and anticipated appropriateness was measured by asking how useful the message would be to them (from ‘not useful at all to me’ to ‘very useful to me’). All surveys included demographic questions. The survey was initially reviewed by a Patient and Public Involvement (PPI) panel and amendments made in response to comments prior to distribution.

#### Procedure

Initial invitations were sent through the database facilitators. Invitations contained an information sheet, an online consent form and a link to a survey. Individuals were screened and consented prior to completing the survey. A paper version of the information, consent and survey were also offered. Participants could withdraw consent (and have their data discarded) up to 1 week after completing the survey.

### Experienced acceptability study (qualitative and quantitative data from a proof-of-concept study)

#### Design

A single group proof-of-concept study explored recruitment, technology feasibility and acceptability of the intervention. Participants initially enrolled for 12 weeks, after this time they were sent a text message asking them to text back ‘stop’ to the system if they did not wish to continue. If the ‘stop’ command was not received, they continued to receive messages for up to another 12 weeks. Participants could provide text ratings of acceptability and were interviewed at the end of the study. A sub-group also provided short interim interviews while receiving messages to identify any technical problems.

#### Recruitment

Participants were recruited by 1) emailing people from Greater Manchester signed up to the RfTF database (as above); 2) asking GP practices in Oxford (*n* = 2) and Greater Manchester (*n* = 1) to search and mail out letters introducing the study to eligible patients; 3) contacting people who had expressed interest in earlier aspects of the development work and had provided consent to be re-contacted about later stages of the project; 4) by installing a pop-up reminder on GP computer systems(*n* = 3) to prompt GPs to provide information at an appointment when a person met the eligibility criteria and 5) by attending a community diabetes education session and providing information about the study.

#### Material development

Separate semi-structured interview schedules were developed by the team for interim and follow-up interviews. Interim interviews focussed on participants’ initial thoughts about the messages and experiences of interacting with the system. Exit interviews explored thoughts about the messages, experiences of using the system and exploring any aspects they particularly liked or disliked. Three of the authors (KB, NN, CK) conducted the interviews and met regularly to discuss the interview schedules and to amend them as needed to further explore areas of interest.

#### Procedure

Potential participants were asked to call or email a member of the research team to express their interest in taking part. A member of the research team then called the person to explain the study and screening questions were asked to ensure eligibility. If eligible, a consent form and prepaid return envelope were sent. A convenient time was also arranged for approximately a week later. A copy of their completed consent was returned to the participant and a copy sent to their GP. Participants were then called as pre-arranged to collect demographic information and participants were enrolled on to the text messaging system.

Participants received between 3 and 4 messages per week and could rate as many messages as they chose according to how easy it was to understand (by texting back ‘EASY’ or ‘HARD’) representing their experienced cognitive response, if they liked it (by texting back ‘LIKE’ or ‘DISLIKE’), representing their experienced emotional response and how useful it was to them (by texting back ‘USEFUL’ or ‘NOT USEFUL’) representing their experienced appropriateness. Once a week, participants were prompted to rate messages with a follow-up message: ‘How did you find the last message?’ with a reminder of the rating options. Participants could also text ‘MORE’ or ‘LESS’ after any message to make receiving a further message from the same BCT either twice or half as likely.

Interviews were conducted when participants stopped receiving messages (either at the end of the study, or if they texted ‘stop’). A small sub-sample also took part in short interim interviews. All interviews were conducted over the telephone, audio recorded and transcribed verbatim. Participants who completed the exit interview were sent a £10 gift voucher as a thank you for their time.

### Analysis

#### Aim 1: comparison of anticipated and experienced acceptability (quantitative data)

Participants’ responses to the survey were coded into numerical values from 1 (lowest point on the scale, e.g., very difficult to understand) to 5 (highest point on the scale, e.g., very easy to understand) for each message. As the survey was newly designed for this project, a Cronbach’s α analysis was conducted to calculate the survey’s internal reliability. Anticipated acceptability ratings were determined by calculating the mean of all participants’ ratings on each of the three items. The mean number of positive (‘EASY’, ‘LIKE’, ‘USEFUL’) ‘and negative (‘HARD’, ‘DISLIKE’, ‘NOT USEFUL’) responses were calculated for each message. Experienced acceptability ratings were then determined by calculating the mean of all responses to each of the three aspects of acceptability (experienced cognitive response (easy vs. hard), experienced emotional response (like vs. dislike) and appropriateness (useful vs. not useful). Pearson’s correlations were calculated to measure the association between the anticipated acceptability score (1–5), and experienced acceptability score (0–1) for each message.

#### Aim 2: assess the applicability of the TFA to a technology-based intervention (qualitative data)

As we wanted to assess the applicability of the TFA to this intervention, a framework analysis approach was taken which permitted the use of an existing framework of codes (the TFA) and the inductive identification of additional codes [16]. Team members familiarised themselves with the transcripts and KB and LM initially double coded 20% of the transcripts with a priori codes based on the seven constructs outlined in the TFA, and researcher-driven codes for data relating to acceptability that did not fit within TFA codes as suggested by the TFA authors [17]. A further 20% of the interviews were double coded (KB & CK) and the coding framework was confirmed during further discussion. The remaining transcripts were then coded based on the coding framework, and the initially coded transcripts were re-checked to ensure nothing had been missed (KB & CK). Coding to the framework was completed individually using NVIVO 12 pro [18] and the individual files merged. The contents of each code were reviewed and a meeting was held to summarise the contents of the codes and identify clusters of codes, patterns between them and potential themes. Mapping and interpretation of the themes was then conducted through several meetings and draft documents with authors.

## Results

### Aim 1: comparison of anticipated and experienced acceptability, results from quantitative data

#### Anticipated acceptability

Out of the 861 people who were potentially eligible, 72 people responded, 61 participants provided some data and those with a less than 10% response rate were removed from the analysis. Fifty-nine participants with type-2 diabetes completed the survey. Participants had been taking tablets for a mean number of 132.4 months (SD = 62.9) and *n* = 18 (31%) had some university level education (see Table 2 for further sample characteristics).

**Table 2:** Sample characteristics

	Anticipated Acceptability study (*n* = 59)	Experienced Acceptability study (*n* = 48)
Mean age (SD)	62.8 (10.8)	63.0 (9.5)
Male n (%)	33 (56%)^a^	33 (69%)
White British n (%)	50 (85%)	43 (90%)
Index of Multiple deprivation decile	Participant’s postcode ^b^	Participant’s GP practice postcode
1–3 (Most deprived)	11 (22%)	13 (27%)
4–7	22 (45%)	26 (54%)
8–10 (Least deprived)	16 (33%)	9 (19%)
Last medication change to diabetes medication^c^		
< 3 months	9 (18%)	9 (19%)
> 3 months but < 6 months	8 (16%)	7 (15%)
> 6 months but < 12 months	6 (12%)	11 (23%)
1 year or over	27 (54%)	20 (43%)

^a^ *n =* 56 as *n =* 3 did not specify ^b^ *n* = 49 for IMD as *n* = 10 postcodes either missing or incorrect ^c^ *n* = 50 for anticipated acceptability survey and *n* = 47 for the experienced acceptability study due to missing data

Messages were rated highest for cognitive response (Mean = 4.23; SD = 0.22), followed by emotional response (Mean = 3.27; SD = 0.23) and finally, lowest on appropriateness (Mean = 2.87; SD = 0.20). All but two of the 72 messages followed this cognitive response > emotional response > appropriateness pattern.

#### Experienced acceptability

In total, 1702 people were contacted about the study, 61 responded, 58 were screened and 48 participants provided consent. Participants were recruited from GP practices with an average Index of Multiple Deprivation (IMD) decile of 5.6 (range 1–10) (see Table 2 for sample information) Two participants withdrew from the study before the initial 12-week completion date. Nine participants chose not to continue receiving messages after 12-weeks. Of the 37 participants who chose to receive messages for a further 12-weeks, 30 completed the full 24-weeks. Participants received messages for a mean of 5.1 months (SD = 1.4). A total of 2025 ratings were received across the 72 messages (Mean = 28.13 per message, Range = 0–78, SD = 21.04). Thirty-one messages were removed from the analysis due to having a less than 10% response rate.

Consistent with the anticipated acceptability data, messages were rated highest on the cognitive response (Mean = 0.99 (SD = .03)), followed by the emotional response (Mean = 0.74 (SD = 0.13). Messages were rated lowest on appropriateness (Mean = 0.68 (SD = 0.14). This pattern was found for 28 of the 42 messages included in the analysis.

#### Comparison of anticipated and experienced acceptability

Two BCTs were identified as outliers in the anticipated acceptability data and removed from the comparison analysis (BCT:10.5_7: Social incentive and BCT: 9.2_8; Pros and cons). Pearson’s correlation analysis was conducted. This revealed a positive association between participants anticipated, and experienced, acceptability scores (r_s_(41) = .422, *p* = .006). The *R*^*2*^ value of .178 indicates a medium to large effect size [19]. When the analysis was conducted on the full set of 72 messages with outliers included, a significant positive correlation was maintained ((r_s_(66) = .376, *p* = .002).

### Aim 2: applicability of the TFA to a technology-based intervention, results from the qualitative data

Thirty-nine participants were interviewed from Greater Manchester and Thames Valley. Interviews lasted between 5 and 30 min. A sub-sample of 16 participants took part in 1–3 interim interviews. In total 22 interim interviews were conducted lasting between 5 and 20 min. Four constructs from the TFA were identified in the data: burden, intervention coherence, affective attitude and perceived effectiveness (see Table 3). We also identified two additional integrative themes which we believe influence participants’ perceptions across the constructs of the TFA: perceptions of appropriateness and participants’ role.

**Table 3:** Results of the qualitative analysis. Definitions of the component constructs in the Theoretical framework of acceptability (TFA) from [2]

Themes identified in the experienced acceptability data	
TFA Construct	Definition
Burden	Experienced burden: the amount of effort that was required to participate in the intervention
Intervention coherence	The extent to which the participant understands the intervention and how it works
Affective attitude	Experienced Affective Attitude: How an individual feels about the intervention, after taking part
Perceived effectiveness	Experienced effectiveness: the extent to which the intervention is perceived to have achieved its intended purpose
**Integrative themes additional to the TFA**	
Perceived appropriateness	The extent to which the individual being interviewed considers the intervention to be applicable and useful for themselves, and the wider population of people with diabetes
Participants’ role	Relates to how difficult it can be during analysis to differentiate someone’s experience of the research from their experience of the intervention
**Constructs in the TFA not identified in the experienced acceptability data**	
Construct	Definition
Ethicality	The extent to which the intervention has good fit with an individual’s value system
Opportunity costs	Experienced opportunity cost: the benefits, profits or values that were given up to engage in the intervention
Self-efficacy	The participant’s confidence that they can perform the behaviour(s) required to participate in the intervention

#### Themes associated with the TFA

##### Burden

Taken literally, the only ‘effort’ required to take part in the intervention was to receive the messages and the majority of people indicated that the system did not seem overly burdensome.

“It's not intrusive, it's not a burden.” Male, aged 67

Participants mainly reported that they thought the frequency of messages (usually 3–4 per week) was about right.

“I like the frequency of the messages. I wouldn’t say that there are loads, there’s not too many to deal with” Female, aged 54

However, a few participants did say they thought the messages were too frequent and some expressed a sense of relief that the study was over. This was often linked to repetitiveness, so where participants felt maybe the medication reminders weren’t necessary for them, they also reported that the messages were too frequent and there was an indication that in this case, the intervention could become burdensome over time.

“I think there were too many…If they'd been different messages...About different topics maybe it would have been OK, but to get these constant reminders of take your tablets, take your tablets, take the tablets”. Male, aged 69

##### Intervention coherence

Regarding understanding the intervention, none of the participants had any problem understanding the concept of the intervention as a text message service for people with diabetes to encourage and support medication adherence and other healthy lifestyle behaviours.

“It’s just a little jolt, isn’t it, to remind you, and generally that will remind you of something else that you should have done or could do” Female, aged 54

However, there were examples of participants being unsure of or unhappy with exactly what the intervention was targeting. Although explained during recruitment, some participants seemed surprised or even irritated that the majority of the messages targeted medication adherence.

“I found it very, very repetitive; they're all regarding taking tablets.” Male, aged 60

Participants also reported that there was little interaction or tailoring of the system so even when they replied ‘more’ or ‘less’ to specific messages these actions seemed to make no noticeable difference.

“I didn’t get the impression that more information was coming at me that was more tailored to what I thought was of benefit.” Male, aged 67

Regarding *how* the intervention worked there was a more mixed experience. The majority of participants reported that the system was easy to use describing it as: ‘straightforward’ ‘simple’ or ‘dead easy’. However, some participants had misunderstandings around interacting with the system, for example trying to provide more detailed answers when only set keywords were recognised, or reporting finding using capital letter cumbersome, despite replies not being case-sensitive.

“Replying to the computer is a no-no as far as I'm concerned. It, you know, it doesn’t sort of make sense to me. But the reminders to take the pills are obviously a good idea.” Male, aged 60

The intervention could therefore be considered in two parts: receipt of messages and interaction. Receiving the messages was coherent and therefore acceptable, whereas the interactive element caused some confusion and frustration amongst a small number of participants and therefore, in its current format, may not be considered acceptable (this was one of the modifications made before beginning a subsequent feasibility trial [20]).

##### Affective attitude

Positive feelings as a result of receiving the messages included describing the messages as: ‘a gentle nudge’, ‘useful’, ‘makes you think’. However, the feelings described tended to be represented by language indicating an absence of negative feelings. For example, ‘wasn’t irritated’, ‘didn’t feel insulted’, ‘didn’t irritate me’.

Negative feelings were expressed with words like ‘condescending’, ‘irritating’, ‘annoying’, ‘frustrating’ and were possibly linked to people feeling they did not need support with taking their medication, or that they already knew most things about diabetes having been diagnosed for a number of years.

“I got three very similar messages about reminding me to take my drugs, and I thought by the third one it was getting a bit patronising.” Male, aged 64

As well as the content of the individual messages, participants spoke about their feelings towards receiving *any* messages about diabetes. For most participants a reminder of their condition was described as positive, to help maintain awareness and motivation to keep doing what they knew they were supposed to be doing.

“Yeah, I think, you know, putting in the odd encouragement, you know, you're doing okay, you get back on track etc, etc. It's psychologically very helpful.” Male, aged 53

However, a few participants mentioned receiving a daily text related to diabetes functioned like an unwelcome daily reminder of their condition, when they would prefer to forget about it.

“it just reminds me that you're not well, you know, it just reminds me that, you know, you've got this condition…sometimes having text messages it also kind of like…puts it into [the] forefront” Female, aged 44

It is probable that participants’ expectations of the system may have influenced their feelings towards it. If a participant was disappointed in the intervention as they had expected more, this could lead them to feeling more negatively towards it.

“I suppose I expected more. I think I expected more interrogation” Male, aged 73

##### Perceived effectiveness

The focus here was on perceived effectiveness for the participant not their suppositions on whether it could be effective for others. Some participants described changes in behaviours directly targeted by the messages, e.g., medication adherence, diet or exercise.

“Well, I now set alarms on my phone to remind me to take [tablets] and think about how I feel when I don’t take them, and it's really... I find it really helpful.” Female, aged 50

Alternatively, some described no changes, and this was often accompanied by the caveat that they did not feel the intervention was appropriate for them, so they felt there wasn’t a lot they needed to change at the outset.

“To be honest, I’m actually quite well organised so not a lot of it was useful for me. It was interesting, but it didn’t change the way I operated shall we say.” Male, aged 68

Some participants reported that rather than the content of the messages, just the act of receiving frequent messages kept their diabetes in mind and resulted in better choices in terms of self-management. This could be explained by increased daily awareness of their diabetes, making ‘slip ups’ less likely or a sense of support from the messages motivating behaviour change as participants described feeling that they ‘were not alone’ and that others were there to help if they need it.

“It's kept my awareness at the level it should be, whereas I think if I hadn’t taken part in the [study] it may have slipped somewhat.” Male, aged 67

### Integrative themes

#### Perceptions of appropriateness

Participants described two aspects of appropriateness: personal and general. The perception of appropriateness over-arches the preceding themes as it influences all participants’ answers.

Participants’ personal circumstances affected the perceived appropriateness of the messages. For example, some participants reported that they took their medication well, and generally managed their diabetes well, and as such, the system as a whole was not relevant for them. For others specific messages did not suit their situation, for example messages around social support when the participant had no family to rely on.

“Some of the things were like, oh, let your children know, or your family know that you mustn’t forget your pills and that. I didn’t find… I mean because of my situation I didn’t find those particularly useful.” Male, aged 66

However, even if the intervention was not perceived as personally appropriate this did not necessarily effect how appropriate participants thought it would be *for others*. For example, positive views of the system were expressed in terms of it being useful and acceptable for others rather than themselves, e.g., people who were newly diagnosed, or those more elderly.

“I can certainly see that it would help other people who haven’t been used to taking tablets”. Male, aged 64

Indeed, the language used to describe who would benefit from the intervention reflected an ‘us/them’ attitude, with many participants referring to ‘others’ who might need support or are poor adherers to their medication.

“You know I haven’t just started [with the medication]. I think that would be the difference and, like I say, I'm relatively healthy and relatively aware of what I need to do. I know of people who perhaps don’t take it so seriously… And it probably is aimed at those people as opposed to me.” Female, aged 48

Whether participants’ perception of themselves as being ‘good at taking their medication’ or managing their blood sugars, was accurate or subject to social desirability bias could not be confirmed, but their beliefs may have impacted on how they engaged with the system and how appropriate they considered it to be. There was acknowledgement that messages that were not seen as appropriate might stick in the mind more than ones that were.

“The ones that stood out the most were the ones that I didn’t think [were] very relevant to me.” Male, aged 66

#### Participants’ role

The interviews indicated variation in the ‘role’ that participants perceived themselves to be taking: some did not view themselves as the target audience, acting almost as co-producers of the system. From initial recruitment to final data collection some viewed their participation as ‘helping’ or ‘giving back’ by supporting research.:

“So, that’s why I do the research things. I don’t expect to get anything specific... I don’t expect they're going to fix this in my lifetime.” Male, aged 61

The role participants saw themselves in came to light particularly when asked why people had continued to receive the messages.

“Well, it seems to me that if I carry on using it, it's not of huge benefit to me, but it might help to refine the systems, more data coming in at your end” Male, aged 67

While this does not directly inform the acceptability of the intervention (aside perhaps from additional information that receiving the texts was not overly burdensome), it could influence the interpretation of the findings. The fact some participants continued to use the intervention as they saw it as their ‘duty’ to do so as research participants has implications on how positively to view continued use as an indicator of acceptability.

## Discussion

The key findings of this study were that for this text message based intervention to encourage medication adherence for people with type 2 diabetes 1) anticipated and experienced acceptability were highly correlated 2) The TFA provided a useful base for understanding acceptability, but that when applying it perceptions of appropriateness and the what role the participant is taking should be taken into account and 3) that taking a mixed-methods approach to understanding acceptability allowed the authors to generate suggestions for measuring acceptability across the lifespan of technology-based health interventions. These findings are further explored below related to each aim.

### Aim1: comparison of anticipated and experienced acceptability

The level of correlation between these two measurements was somewhat surprising and suggests in some cases that anticipated acceptability could be used instead of measuring experienced acceptability. However, there was a difference seen in the consistency of the pattern of responses between the two studies. In both studies, messages were rated most highly on the cognitive facet, then emotional, then appropriateness; however, this was found more consistently in the anticipated acceptability results (70/72 messages) than the experienced acceptability results (28/42 messages). This could indicate those receiving the messages liked or understood them less than those reading them on a survey. The pattern found may be useful when developing acceptability assessments, and when appraising how studies have measured acceptability. If measures/ questions only take into account cognitive aspects (was it easy to understand?) or emotional aspects (was it liked?) this could give a more positive result than if they consider how appropriate participants felt the intervention was for them.

### Aim 2: the utility of the TFA in the context of a technology-based healthcare intervention

The TFA proved to be a useful base to begin analysing the qualitative data. Four of the seven constructs were identified as themes within this data. Using text messages to deliver the intervention meant opportunity costs and self-efficacy were unlikely to be key constructs in this case. The cost of receiving and/or replying to text messages is low and all participants had some familiarity with the medium so, self-efficacy (as defined in the TFA) to ‘perform the behaviours required to participate in the intervention’ would have been high. The participants in this case were convinced of the value of medication, and an intervention to support taking it as prescribed, so ethicality was not identified as a theme here. The analysis identified two additional themes, perceptions of appropriateness, and participants’ role. Perceived appropriateness is included in the definition of acceptability given by Sekhon et al. [2], but the level of influence seen in this qualitative data suggests to us that it may be a useful explicit addition to the TFA. Exploring participants’ role as part of the analysis highlighted the potential effect the context of taking part in research, and speaking to researchers may have had on the overall experience of using the intervention. That is, it possibly increased the reported acceptability of the intervention despite it not being perceived as appropriate for a proportion of participants. This could be due to participants’ being keen to help others with diabetes, or a result of social desirability bias with participants wanting to appear as ‘good’ self-managers who do not need help with medication adherence. If implemented, it is more likely if an intervention is not thought to be appropriate that people would not use it. Therefore, although anticipated and experienced acceptability were found to be correlated, this finding indicates that as the purpose of acceptability testing may change, the key constructs to measure will also change.

### Aim 3: suggestions for measuring acceptability of technology-based health interventions over the lifespan of development

Considering the qualitative and quantitative findings together led to three suggestions for measuring acceptability in technology-based interventions:

1. Specifically ask about, or measure perceived appropriateness as this may influence other facets as well as being a product of them.

Identification of perceived appropriateness as a theme that can influence the other TFA constructs, and the fact that appropriateness was often scored below cognitive and emotional response of acceptability suggests that, rather than appropriateness being based on these responses (as described in the Sekhon et al. [2] definition and shown in Fig. 1), there is a bi-directional relationship, and that these emotional and cognitive responses are also affected by perceptions of appropriateness.

2. Identify whether the acceptability measured relates to the intervention itself (or participation in research) and whether it concerns the individual being asked (or the individual is answering as a representative of a group).

The qualitative data gave important insights into how participants might be answering questions related to acceptability. The quantitative results may have been affected by individuals answering generally for people with type 2 diabetes, rather than answering directly for themselves. In addition, continued use of the intervention may not reflect future use outside of a research study as participants may have found being a participant in the research acceptable, but would not use the intervention if they were offered it through their general practice.

3. Consider what facets of acceptability are most important at which point in the development lifespan of the intervention and how to use qualitative, quantitative or mixed methods data to best understand these.

The correlation between anticipated and experienced acceptability may indicate that not all facets of acceptability need to be measured throughout. In the initial stage of development, anticipated acceptability may be measured to ensure an intervention is not unacceptable. As the intervention develops through piloting, feasibility, efficacy trial and implementation the purpose of assessing acceptability may change from ensuring it is not unacceptable, to predicting future use of, or satisfaction with the intervention. If perceived appropriateness is not found in the latter stages, the intervention may not be unacceptable, but it may not be used.

### Strengths and limitations

Using a mixed methods approach and comparing a study of anticipated and experienced acceptability has provided an in depth understanding of the acceptability of this intervention. This can be used to further refine the intervention, and can be applied by others when designing and appraising technology-based, health related interventions. To our knowledge, this is the first paper to compare anticipated and experienced acceptability of the same intervention. We used different populations of participants for the survey and the proof-of-concept study. The same pattern was found and the results were correlated, but the differences in consistency of the pattern cognitive responses > emotional responses > appropriateness could be due to differences in the two populations, rather than differences between anticipated and experienced acceptability. For example, level of education may have influenced how easy the messages were to understand, but this data was only collected for the anticipated acceptability survey so cannot be compared. To investigate whether this is the case a longitudinal design would be needed to assess acceptability over time within participants.

Our recruitment strategy resulted in a response rate of 7% and 3% for the anticipated acceptability survey and experienced acceptability study respectively. Although fairly typical of recruitment for diabetes studies through database search and invitations [e.g. [21, 22] this may have an influence on how generalisable the findings are to the wider population of people with type 2 diabetes. The recruitment procedure may have resulted in a larger proportion of people who were satisfied and confident with their diabetes self-management than the general population of people with diabetes. The majority of participants in both studies had been taking their medication without changes for the last 6 months or more. The expansion provided by the qualitative work described here is helpful to understand the influence this may have had. In populations who were less confident, or newer to taking medication, the perceived appropriateness may have been higher, and therefore the distinction between appropriate for me and appropriate for others may be less important. In addition, the participants’ role theme may be less present in other populations as the main reason for taking part in the intervention may be more to do with the medication adherence content than the research itself. In highlighting these issues, it is hoped that future researchers can be aware of how these may influence acceptability findings. The majority of the sample for both studies were male and White British, despite recruiting from GP practices with diverse populations. This could limit the generalisability of these findings in terms of how acceptable these messages may be for more diverse populations. This work was conducted within a larger project that has included studies specifically recruiting South Asian populations [23], due to the increased risk of diabetes within this population [24], similarities and differences between findings will be considered in the future with the aim of developing an intervention with appeal to a broad range of people with type 2 diabetes. =.

## Conclusions

Understanding acceptability across the lifespan of an intervention could inform further development of an intervention to improve acceptability. The positive correlation found between anticipated and experienced acceptability suggests that gaining a better understanding of acceptability at an early stage, before an intervention is used, would allow for improvements to be made prior to conducting more expensive feasibility studies. Considering the reasons for measuring acceptability at each stage, for example to ensure an intervention is not unacceptable or to predict future use will determine which constructs are most important to measure, and when. The TFA provided a good framework for this analysis but the importance of perceived appropriateness could have been missed without additional inductive coding. Although included in the definition of acceptability, the qualitative data presented here suggests that rather than being ‘based on’ cognitive and emotional responses, perceived appropriateness has a role in influencing these responses suggesting a two-way relationship. Finally, when measuring or evaluating the reported acceptability of an intervention, an understanding of whether participants are responding to the acceptability of the intervention or the research, and whether they are commenting on acceptability for themselves or others is important.

## Data Availability

The quantitative dataset used and/or analysed during the current study are available from the corresponding author on reasonable request. The qualitative dataset is not available as consent to share this data was not requested from participants.

## Abbreviations

BCT: Behaviour Change Technique
RftF: Research for the Futures
SuMMiT-D: Support through Mobile Messaging and digital health Technology for Diabetes
TAM: Technology Acceptance Model
TFA: Theoretical Framework of Acceptability
